# Transgenic Potatoes for Potato Cyst Nematode Control Can Replace Pesticide Use without Impact on Soil Quality

**DOI:** 10.1371/journal.pone.0030973

**Published:** 2012-02-16

**Authors:** Jayne Green, Dong Wang, Catherine J. Lilley, Peter E. Urwin, Howard J. Atkinson

**Affiliations:** Centre for Plant Sciences, University of Leeds, Leeds, United Kingdom; Ghent University, Belgium

## Abstract

Current and future global crop yields depend upon soil quality to which soil organisms make an important contribution. The European Union seeks to protect European soils and their biodiversity for instance by amending its Directive on pesticide usage. This poses a challenge for control of *Globodera pallida* (a potato cyst nematode) for which both natural resistance and rotational control are inadequate. One approach of high potential is transgenically based resistance. This work demonstrates the potential in the field of a new transgenic trait for control of *G. pallida* that suppresses root invasion. It also investigates its impact and that of a second transgenic trait on the non-target soil nematode community. We establish that a peptide that disrupts chemoreception of nematodes without a lethal effect provides resistance to *G. pallida* in both a containment and a field trial when precisely targeted under control of a root tip-specific promoter. In addition we combine DNA barcoding and quantitative PCR to recognise nematode genera from soil samples without microscope-based observation and use the method for nematode faunal analysis. This approach establishes that the peptide and a cysteine proteinase inhibitor that offer distinct bases for transgenic plant resistance to *G. pallida* do so without impact on the non-target nematode soil community.

## Introduction

Arable agriculture must remain productive and become more sustainable than in past decades [1]. A key aspect of this is maintaining soil quality for which soil organism abundance, diversity, food web structure or community stability are useful indicators and important contributors to soil function. They are responsive to land management practices [2] and are of value in defining when soil organisms have been exposed to harm. The European Union (EU) seeks to protect soils and their biodiversity [3] for instance by changes to its directive on use of plant protection products [4] to reduce usage of those pesticides that harm soil quality. An example consequence is that the pesticides currently applied to 23% of UK potato fields to control potato cyst nematode (PCN) may be withdrawn either abruptly or progressively thereby doubling the economic cost of this pest, in the UK alone, to £56 m/year [5], [6]. This is a serious concern as one of the two species of PCN, *Globodera pallida*, is particularly difficult to control by either crop rotation or the partially resistant potato cultivars available against it [7]. One future economic control option is genetically modified, nematode-resistant (GMNR) potato cultivars but the potential of such crops to provide essential pest management and reduce pesticide usage is inadequately considered in current EU policies. Each transgenic trait/crop developed must be shown to be both effective and environmentally benign under field conditions before its deployment can be considered. The expression of a plant cysteine proteinase inhibitor (cystatin) in potato roots controls potato cyst nematode (*Globodera* spp) by impairing digestion of its dietary protein [8]–[10]. Growth of these GMNR plants did not harm above ground organisms [11], [12] or soil microarthropods [13] in potato fields. A small effect on the soil microbe community was detected by phospholipid fatty acid analysis in some years [13] but not others [14]. It was insufficient to influence soil function as assessed by litter decomposition and was much less than imposed by seasonal factors like soil moisture content. A second sensitive approach (community level physiological profiles) that measures substrate use by rhizosphere bacteria did not identify changes imposed by the cystatin-expressing transgenic potato plants but readily detected the consequences of growing different conventional crops [15].

Our work to develop GMNR potato plants, test their efficacy and evaluate their possible impact on biotic aspects of soil quality has now advanced in three ways. First, we report the efficacy of a second GMNR trait in providing field control of *Globodera* spp in addition to the cystatin used in previous work. The new potato plants secrete a non-lethal peptide from their roots that disrupts the chemoreception that cyst nematodes require to locate host plants [16]. The peptide was originally obtained by biopanning a phage display library against membrane fractions of *Caenorhabditis elegans* that are rich in nicotinic acetylcholine receptors (nAChR). The peptide was displaced by the anthelmintic levamisole that binds to these receptors [17]. It is a disulphide-constrained 7-mer, termed nAChRbp [18] with the amino acid sequence CTTMHPRLC that inhibits chemoreception of *G. pallida* at 1 µM [19] and reduces parasitism of hairy roots of potato [16]. Fluorescent tagging has shown the peptide is taken up from an aqueous environment by the primary cilia of some nematode chemoreceptive sensilla before undergoing retrograde transport to their neuronal cell bodies. It is then transported to a limited number of connecting neurons and probably exerts its effect at the synapses of cholinergic interneurons [18]. The second new aspect aimed to enhance biosafety of the transgenic approach by restricting expression of the peptide to root tips using a tissue-specific promoter (*MDK4–20*) that we have previously described from *Arabidopsis* [20]. The third development was to improve an approach that detects the direct or indirect impact of the transgenic lines on non-target soil nematodes. Nematodes are worthy of such consideration as the most abundant metazoan taxon with a high abundance in soil where their community participates in many functions at different levels of the soil food web. These nematodes are stable in response to fluctuations in moisture and temperature while responding to land management effects in predictable ways that reflect changes in soil microenvironments [21]. Soil nematode faunal analysis has been based on a weighted abundance for five sub-groups derived from the rapidity of their multiplication in favourable conditions [22], [23]. One outcome is an enrichment index (EI) ranging from 0–100 for nematodes that respond rapidly to environmental change and a structural index (SI) with the same score range for those that prefer undisturbed habitats. These indices enable the extent of soil disturbance, enrichment, the decomposition channels, C:N ratio and food web condition to be inferred [23]. One drawback to nematode faunal analysis is the requirement for time-consuming microscopic identification of nematode genera in many samples, a technique that relies on skilled assessment of a range of morphological features. We have overcome this potential obstacle to rapid analysis by deploying a molecular bar-coding approach based on the 18S ribosomal gene [24], [25] to recognise nematode genera in mixed samples. Use of that method suggests no impact on the nematode faunal index and hence soil health when GMNR potato plants are deployed to control *Globodera*.

## Results

### Transgenic potato lines secreting a chemodisruptive peptide

**Figure 1:**
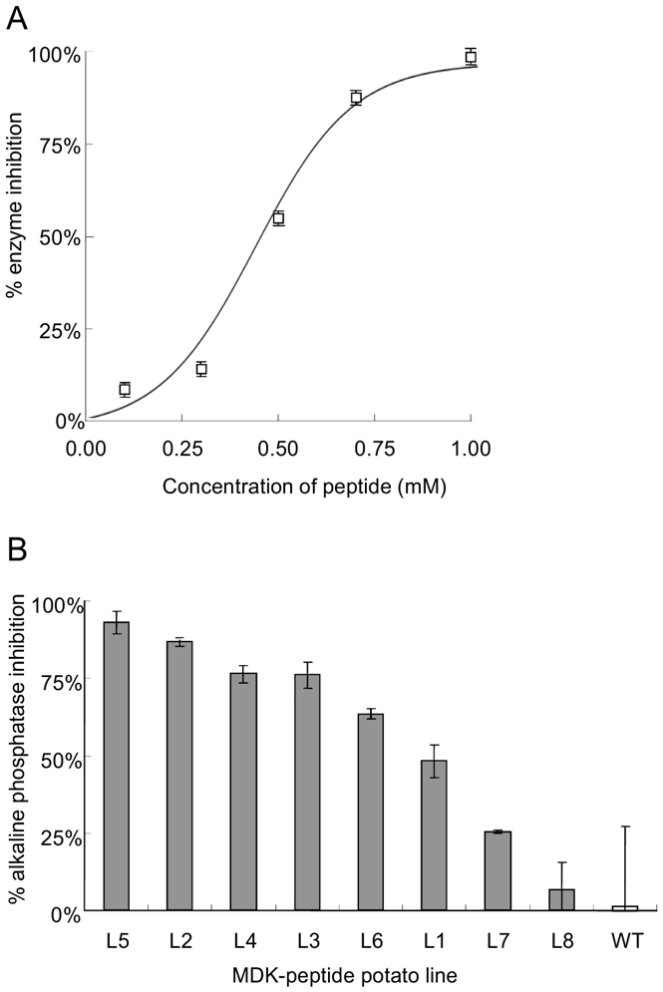
Inhibition of alkaline phosphatase by the behavioural disrupting peptide. (A) The chemically synthesised peptide inhibited alkaline phosphatase in concentration dependent manner. (B) Sufficient peptide could be collected from root exudates of transgenic potatoes to inhibit the enzyme. Values are means ± sem.

Forty transgenic cv Désirée potato lines were generated that were confirmed by PCR to contain the MDK-peptide construct (results not shown). The peptide has previously been shown to inhibit alkaline phosphatase [16]. The effect is concentration dependent with 1 mM of the synthetic peptide providing almost complete inhibition of the enzyme in the experimental assay conditions (Figure 1A). Root exudates of all transgenic lines were screened for their ability to inhibit alkaline phosphatase in comparison to exudates from wild-type plants. Exudates from several lines caused detectable inhibition and data for a range of these lines are presented in Figure 1B. Comparison with the standards suggests that up to 860 µM of the peptide accumulated in root exudates after 14 days incubation of the plant in 7 ml of water. Three MDK-peptide potato lines (L1–3) were selected for further evaluation on the basis of their level of active peptide secretion and normal growth phenotype.

### Cyst nematode resistance conferred by the secreted peptide

**Figure 2:**
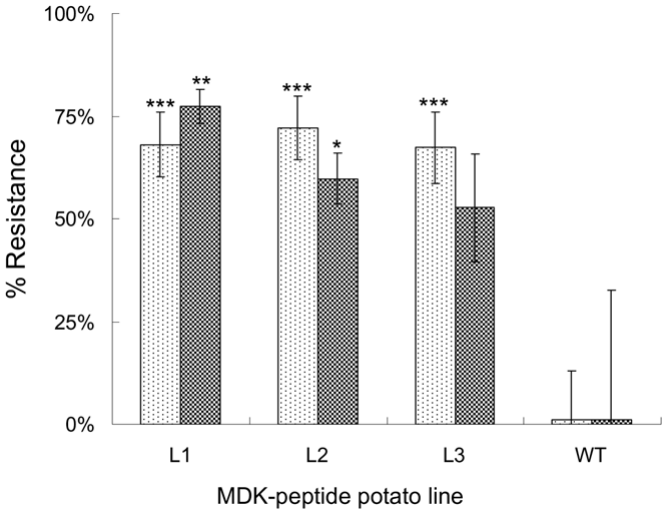
Resistance to *Globodera pallida* provided by transgenic expression of a behavioural disrupting peptide in potato. Resistance is conferred by expression of the peptide under control of a root-tip specific promoter (*MDK4–20*) in trials in both a containment glasshouse (light shading) and the field (darker shading) relative to *G. pallida* eggs in soil after multiplication on untransformed cv Désirée. Values are means ± sem based on 2 replicate samples for each of ten pots per line in containment and pools of nine samples for each of 4 replicate plots per line in the field. The differences between transgenic lines and the corresponding wildtype control are shown (***, P<0.001; **, P<0.01; *, P<0.05; oneway ANOVA with *a priori* contrasts).

The three selected potato lines were challenged by *G. pallida* in a containment glasshouse trial before promising results led to them being advanced to a replicated field trial. There was concordance between resistance observed in the containment and field trials with transgenic lines supporting significantly reduced nematode multiplication compared to wild type plants in both environments. Up to 77±4% (mean ± sem) resistance was obtained for the most effective line in the field (Figure 2). A general linear model with univariate analysis on transformed arcsin values established significant difference between transgenic and control plants (P = 0.001) but not between the two environments (P = 0.9) and there was no significant interaction between the two factors (P = 0.089). Oneway ANOVA with *a priori* contrasts established each peptide-expressing line differed from the untransformed wildtype plants in containment (P<0.001 in all three cases) and for two lines (L1 and L2) in the field trial (P<0.01 and P<0.05 respectively).

The initial containment trial to assess resistance did not include plants expressing the cystatin OcIΔD86 under control of either the constitutive CaMV35S promoter or the ARSK1 promoter expressed preferentially at feeding sites of cyst nematodes. This is because the efficacy of these particular transgenic lines in containment and field trials has been reported previously [9], [10]. The level of resistance in the field in the current work was 57±13% for ARSK/OcIΔD86 plants and 53±7% for CaMV35S/OcIΔD86 plants. Potato cv Santé, which has natural resistance to *G. pallida*, provided a resistance level of 96±1% in the field compared to the wild type, fully susceptible cv Désirée. Populations of *G. pallida* vary in their virulence to cv Santé, with lower levels of relative resistance recorded on previous occasions [9].

### Development of genus-specific qPCR primers for detection of soil nematodes

The 18S small subunit ribosomal RNA gene (SSU) of nematodes is approximately 1600 bp in length. The 500–600 bp region at the 5′ end contains both conserved stem and more divergent loop structures, accounting for around half the nucleotide variability of the complete gene [25]. Sequencing of this DNA region, amplified using a standard primer pair, enabled individual nematodes to be assigned a likely genus by cross-reference to database sequence entries.

**Figure 3:**
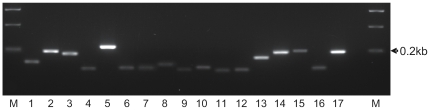
PCR products amplified using genus specific primers. The template for each pair of primers was DNA extracted from two hundred nematodes of mixed genera as recovered from the field site. The primers, detailed in Table 2 for non-parasitic genera, were designed to amplify SSU sequence from (1) *Acrobeloides/Cephalobus* (2) *Anatonchus*, (3) *Aphelenchoides*, (4) *Aporcelaimellus*, (5) *Coslenchus*, (6) *Ditylenchus*, (7) *Eucephalobus*, (8) *Filenchus*, (9) *Globodera*, (10) *Helicotylenchus*, (11) *Mesodorylaimus*, (12) *Pellioditis*, (13) *Pelodera*, (14) *Rhabditella*, (15) *Scutylenchus*, (16) *Ceratoplectus* (17) *Anaplectus*. (M) Hyperladder I marker.

Ninety three nematodes were randomly sampled during an initial survey to identify those soil nematodes present at the field site prior to planting. Each individual was assigned a tentative genus based on morphological identification, although it was not possible to distinguish between the morphologically similar *Pellioditis* and *Pelodera* or between *Acrobeloides* and *Cephalobus*. Sequence of the 18S SSU gene was obtained for 96% of the nematode individuals sampled and this revealed the presence of 21 genera. The preliminary morphological identification of each nematode was confirmed by comparison of the sequence obtained from its PCR product with database entries (Table 1). The sequence analysis allowed discrimination of *Pellioditis* and *Pelodera* so that genus specific primers could be developed for these two nematodes. It was not possible to discriminate *Acrobeloides* and *Cephalobus* individuals in the sample by either observation or sequence comparison but this distinction was unimportant for the current work as they belong to the same functional guild. Each sequence generated was 99–100% identical to database entries for a single genus thus allowing unambiguous genus assignment except for four individuals with sequence that was only 95% identical to database entries for *Aphelenchoides* as the most similar genus. Morphological identification also assigned these nematodes to the *Aphelenchoides* genus and they were considered as such for this work. Eight of the genera identified were plant feeding nematodes that are not included in nematode community index calculations. The sequences of each genus other than those of plant parasites were used to design 11 primer pairs (Table 2) that would each be specific for a particular genus and suitable for use in quantitative polymerase chain reaction (qPCR). The 11 primer pairs allowed detection and discrimination of >90% of the non-plant feeding nematodes identified at the field site. Sequence divergence between the genera was sufficient to allow two unique primers to be used in seven cases. It was not possible to design unique primer pairs that also satisfied the desired criteria for qPCR for the remaining four genera *Anaplectus*, *Mesodorylaimus*, *Eucephalobus* and *Acrobeloides/Cephalobus*. For specific detection of these genera, one common forward primer (GUF2) was used in combination with a unique reverse primer. All primer pairs were tested initially in a standard PCR using template DNA from single nematodes of the corresponding genus and subsequently using DNA prepared from a random pool of 200 nematodes from the field site (Figure 3). A single, unambiguous sequence matching the expected genus was obtained for each product amplified from pooled DNA, confirming the specificity of the primers. In qPCR, dissociation curves exhibited identical single peaks when using template DNA from either single or pooled nematodes. The final verification of the specificity of the primers centred on discrimination achieved by the qPCR primer pairs for *Acrobeloides/Cephalobus* and *Eucephalobus* which have highly similar SSU gene sequences. No cross amplifications were detected when *Eucephalobus* specific primers (GUF2/EuR1) were used for amplification from *Acrobeloides/Cephalobus* genomic DNA, or when *Acrobeloides/Cephalobus* specific primers (GUF2/AcCR2) were used on *Eucephalobus* DNA. In both cases no threshold cycle (Ct) value was recorded using template from non-target species after 40 PCR cycles in contrast to Ct values of <30 cycles for the target sequences (Figure S1).

**Table 1:** Free-living soil nematode genera recovered at the field site.

Genus	Functional guild*	Sequence identity (%)	NCBI accession
*Acrobeloides/Cephalobus*	Ba_2_	100	AF430515, AF034390
*Anaplectus*	Ba_2_	99	AY284696
*Ceratoplectus*	Ba_2_	100	AY284706
*Eucephalobus*	Ba_2_	100	AY284667
*Aphelenchoides*	Fu_2_	95	DQ901551
*Pellioditis*	Ba_1_	99	AF430633
*Pelodera*	Ba_1_	99	AF083002
*Rhabditella*	Ba_1_	100	AY284654
*Anatonchus*	Ca_4_	99	AJ966474
*Mesodorylaimus*	Om_4_	100	AJ966488
*Aporcelaimellus*	Om_5_	100	AY284812

*Ba, bacterivore; Fu, fungivore; Ca, carnivore; Om, omnivore.

These genera contributed >90% of individual non-parasitic nematodes at the site. The functional guild and their position on the coloniser-persister scale (1–5, shown as a suffix) are as previously assigned to each genus [22], [23]. The sequence identity of the amplified region of 18S small subunit ribosomal RNA gene to that of the most similar genus in the GenBank data set is indicated together with the Accession Number of the most similar database sequence.

**Table 2:** Primer pairs used in qPCR to identify each nematode genus.

Genus	Primer*	Primer sequence	Product (bp)
*Aphelenchoides*	AphF	TTGGACTGCCATGGTGTTGA	173
	AphR	ATGTCCGACCTCATAGAGAAC	
*Pellioditis*	PellF	AAGTTTTCGGCTGCCTTTTAG	161
	PellR	ACAGTTTACGGCCATCGGAA	
*Pelodera*	PeoF	GAACGCCGTTTCGGTTTTTC	189
	PeoR	AAGCTAACGCTCGGTTTCATA	
*Rhabditella*	RhaF	TTTTACCTATTCCGAAATCTTATT	198
	RhaR	TGGTTGATAGGGCAGACTCC	
*Anatonchus*	AnF	TGGTAAGAATTGGTAAACACGA	188
	AnR	CGAGTCCAGTCCGAAGAATT	
*Aporcelaimellus*	ApoF	GTTACGCCTAGTTCGGAAGA	222
	ApoR	GCACTCATTACAAGCACCTTT	
*Ceratoplectus*	CplecF	GGTAAACCCCTCAAAATCCTA	190
	CplecR	GAAAACCCCGACAGCAGCA	
*Acrobeloides/Cephalobus*	GUF	TTACGTCCCTGCCCTTTGTA	121
	AcCR	TCAAATCAGTTTCCAGCGAAC	
*Anaplectus*	GUF	TTACGTCCCTGCCCTTTGTA	93
	AnaplR	CCCGAAAGCCCCAACAGC	
*Mesodorylaimus*	GUF	TTACGTCCCTGCCCTTTGTA	95
	MeR	GCACTTTCGTACACCTTAACT	
*Eucephalobus*	GUF	TTACGTCCCTGCCCTTTGTA	118
	EuR	AGTCAGCTTCCAACGACTCG	

*F, forward primer; R, reverse primer.

Once the specificity of the primers for the nematode community under study was determined, it was necessary to relate the quantity of product from qPCR to that for a single nematode of each genus. This was achieved in two steps by first using the mean DNA content per nematode from qPCR analysis with *Aphelenchoides* as an arbitrary calibrator. A subsequent minor adjustment was then made from relating parallel morphological counts to qPCR-based abundance data of mixed nematodes in replicate soil samples.

### Validation of the qPCR measurement of nematode faunal indices

The qPCR-based method of determining nematode faunal indices was validated using soil samples taken from the field trial.site in autumn following a potato crop. Nematodes were extracted from multiple sub-samples from each of three plots for one determination of genera present by microscopic examination of morphology and three to seven replicate analyses by qPCR per plot. The proportion of each genus identified by the two methods is presented in Table 3 and the derived EI and SI values are shown in Table 4. The qPCR approach distinguished between the morphologically very similar *Pellioditis* and *Pelodera* that were grouped together for results of morphological analysis. The two genera belong to the same functional guild so inability to distinguish between them morphologically does not influence the indices derived in this work. The percentage sample compositions recorded by the two approaches were similar for the prevalent genera with the mean from qPCR not differing significantly from the corresponding value from morphology (single sample t tests). *Pellioditis* and *Pelodera* together comprised 74±1% of the non-plant parasitic nematodes as determined by morphology compared with a value of 78±5% from qPCR analysis. The corresponding values for *Acrobeloides*/*Cephalobus* were 24±1% by morphological analysis and 20±5% by qPCR. Values for genera present at only a very low abundance are likely to vary between samples by both chance and as a result of a patchy nematode distribution. The full set of 11 primer pairs was used for analysis of these test samples by qPCR, but some genera were not detected either by microscopic examination or by qPCR. *Anaplectus* and *Ceratoplectus* were present in the initial soil sample and so primers had been developed for these genera. They were not recorded in subsequent field samples analysed by either the morphological or the qPCR-based approach. *Rhabditella* was also present in the initial sample and had a low abundance in soil used for the containment trial but was not detected in subsequent field samples.

**Table 3:** Proportions of nematode genera determined by both morphology and qPCR for field soil samples.

		Soil sample 1		Soil sample 2		Soil sample 3	
Genus	Functional guild	morphology	qPCR	morphology	qPCR	morphology	qPCR
*Pellioditis*	Ba_1_	73%	74±5%	75%	61±15%	75%	47±11%
*Pelodera*	Ba_1_		0%		8±8%		36±14%
*Aphelenchoides*	Fu_2_	1%	0%	1%	0%	1%	0%
*Acrobeloides or Cephalobus*	Ba_2_	25%	25±5%	22%	28±18%	24%	15±5%
*Eucephalobus*	Ba_2_	0%	1±1%	0%	3±1%	0%	1±0.4%
*Anatonchus*	Ca_4_	0%	0%	1%	0%	0%	0%
*Mesodorylaimus*	Om_4_	2%	0%	1%	0%	0%	0%
*Aporcelaimellus*	Om_5_	0%	1±0.2%	0%	0%	0%	0%

The percentage of each nematode genus present was estimated from three field soil samples using in each case one sub-sample for morphological identification and 3–7 sub-samples for qPCR-based analysis. The functional guild and their position on the coloniser-persister scale (1–5, shown as a suffix) are as previously assigned to each genus [22], [23]. The percentages are based on the number of nematodes observed (morphological analysis) or on all nematodes (>200) extracted from each 100 g sub-sample used to prepare template for qPCR. *Anaplectus*, *Ceratoplectus* and *Rhabditella* were not detected by either morphology or qPCR in these samples. Values for qPCR represent means ± sem.

**Table 4:** Values for enrichment (EI) and structural (SI) indices of soil nematode communities determined by both morphology and qPCR.

	Soil sample 1		Soil sample 2		Soil sample 3	
	morphology	qPCR	morphology	qPCR	morphology	qPCR
EI value	92.0	92.1±2.3	92.9	89.8±8.89	92.5	95.2±1.71
SI value	26.7	13.0±2.76	17.4	1.3±1.48	0.0	5.0±1.53
No. of nematodes	175	>200	166	>200	106	>200

The functional guilds of each genus [22] and their standard weightings were used to calculate the components of the food web from which the enrichment (EI) and structural (SI) indices were calculated for each soil sample (see [23] for further details). Values derived from qPCR data represent means ± sem.

### Determination of EI and SI values by qPCR during containment and field trials

**Figure 4:**
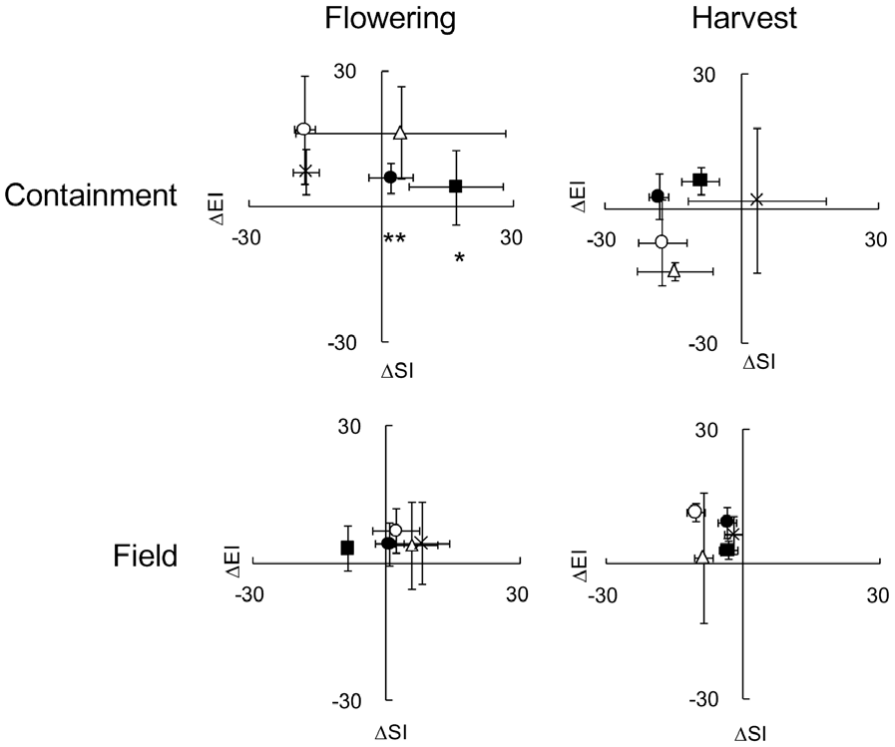
Changes in enrichment and structural indices for soil nematode faunal analysis relative to pre-plant levels. The changes for enrichment (ΔEI) and structural indices (ΔSI) were measured in both containment and the field at potato flowering and immediately before harvest. For each trial, soil was sampled from around the roots of oil seed rape (○), untransformed potato cultivars Santé (**Δ**) and Désirée (**X**) plus 2 lines of the latter cultivar expressing a cystatin [9], [10] (▪) and 3 lines expressing the peptide (•). The containment trial involved three pots for each plant type. For the field trial there were nine pooled samples for each of four replicate plots/line or plant type. Values are means ± sem.

Soil was collected from the site of the future field trial for use in a containment trial set up to assess effects of the transgenic lines on the soil nematode community. Included in this trial were transgenic potato lines expressing either a cystatin or the chemodisruptive peptide, wild-type cv. Sante and Désirée and oil seed rape (OSR) plants. The nematode faunal analysis for the containment soil prior to planting gave an EI = 48.0±9.8 and SI = 23.1±9.2. Differences (Δ) from these initial values are given for each type of plant, at either flowering time or harvest, in the upper panels of Figure 4. The univariate procedure of the general linear model suggested that ΔEI but not ΔSI varied overall between the flowering and harvest samples (P = 0.016 and P = 0.11) but neither index varied among lines. Further analysis used oneway ANOVA with *a priori contrasts* to compare ΔSI values for untransformed cv. Désirée with those for transgenic lines. This suggested soil that supported growth of OcIΔD86 cystatin and peptide expressing plants had higher SI values at flowering (P = 0.023 and 0.006 respectively) than untransformed cv. Désirée whereas at harvest there were no significant differences between the soil samples (P>0.28 in all cases).

The detection of a difference with sampling time and some differences between lines at one of the two sampling times encouraged us to use this approach in a field trial in the following spring. On this occasion, the values at planting were EI = 80.3±6.1 and SI = 9.3±1.5. Differences (Δ) from these initial values are presented for each type of plant, at either flowering time or harvest, in the lower panels of Figure 4. In this trial, the univariate procedure established a significant difference between flowering and harvest in ΔSI (P = 0.021) but not for ΔEI (P = 0.28). No significant differences were evident by this statistical approach between lines for either index (P = 0.61 and P = 0.22 respectively). Analysis for the field trial was developed further using oneway ANOVA with *a priori contrasts* as in the containment trial. This detected no differences in ΔEI or ΔSI values of transgenic lines relative to untransformed cv. Désirée at either sample time. There were also no significant differences between untransformed Désirée and OSR which was chosen as the non-solanaceous comparison crop because it is grown in rotation with potato at the field site. The lack of any differences between untransformed and transformed cv. Désirée at flowering or harvest establishes little impact of the transgenic plants on the non-target nematode soil community in the field in spite of the level of control of *G. pallida* that was achieved.

## Discussion

Potato plants were developed that transgenically expressed a disulphide-constrained peptide (nAChRbp) capable of binding to nematode acetylcholine receptors and inhibiting chemoreception of cyst nematodes. A tissue-specific promoter restricted expression of the peptide to the outer cell layers of the root tip. Exudates from the transgenic potato plants inhibited alkaline phosphatase as expected if the peptide was successfully expressed and secreted from the roots. However it was uncertain that the quantity of peptide secreted into soil and its stability there would ensure effective resistance when transgenic potato plants were challenged with *G. pallida*. The potato lines trialled did display a level of resistance to *G. pallida* in both a containment glasshouse trial and in the field that establishes the potential of this novel approach.

The results validate the root tip-specific promoter of the *Arabidopsis MDK4–20* gene [20] as a means of delivering effective root protection by the peptide under field conditions. This promoter is active in potato in both the zone of elongation and root border cells even after they detach from the root cap, often decorating the zone of elongation. This may enhance protection of this zone from invading nematodes [20]. PCN normally invades near root tips which slows root extension, particularly by lateral roots. This reduces the volume of soil from which the plant draws water and nutrients [26]. The peptide's mode of action suppresses this important aspect of the pathology before other defences such as a cystatin could act as an anti-feedant on just those nematodes that establish in roots. This suggests the resistance conferred on potato roots by expressing these different traits should be additive. If so, this is likely to prevent economic damage by *G. pallida*. Both a cystatin [10] and the peptide have provided >75% resistance so if fully additive they should provide circa 95% control. This possibility will be studied in future work.

Plants expressing the peptide-based resistance, or a previously described approach involving transgenic expression of a cystatin in nematode feeding cells, had no impact on standard enrichment or structural indices of the non-target nematode soil community relative to changes caused by non-transgenic potato plants. The relative abundance of nematode genera that contributed to the faunal indices was determined by qPCR analysis of DNA from pooled nematodes extracted from soil samples. This employed genus-specific primers designed from 18S SSU DNA sequence of those nematodes present at the study site. The values obtained by determining EI and SI concurrently for replicate samples by morphology and the qPCR approach established that the molecular technique provided reliable estimates of these indices. This outcome is consistent with nematodes being particularly suitable for a normalised qPCR approach for determining the relative abundance of each genus. Their somatic cells are post-mitotic and growth involves an increase in cell size rather than number [27]. In *C. elegans* the genome copy number rises 2–4 fold as germ line cells increase during adult development [28] before this new level is maintained [29], [30] until a post-reproductive variation in DNA content occurs [28]. Errors associated with variation in the relative abundance or reproductive state of adults in soil samples were clearly unimportant in the current work given the good agreement obtained with the estimates based on morphological identification.

Measurements of faunal indices were made at flowering when the root size of potato plants peaks [31] and immediately prior to harvest. The current work emphasised changes (ΔEI and ΔSI) relative to pre-plant values to compare the relative effect that the different plantings imposed rather than absolute values that reflect past agricultural activity. The SI value is primarily determined by omnivorous and predatory nematodes that are sensitive to disturbance. They are often uncommon and variable in frequently tilled arable soils [32] such as those used in this work. The fall in SI value between the two pre-plant samples taken for the containment trial and the later field trial probably reflects the impact of tilling which occurred just before establishing the field trial in spring. It is the more tolerant taxa contributing to SI that are likely to persist in such conditions [33]. In contrast, soil for the containment trial was collected in the summer of the previous year. The ΔSI values associated with the transgenic lines were greater at flowering in the containment trial only and no other differences from soil supporting growth of untransformed Désirée were recorded. The EI value is a good indicator of the amount of N in the soil [34] as it detects changes for those genera that respond to increased bacterial abundance arising from factors like fertiliser application and root turnover. The growth of GMNR lines in soil had no significant adverse effects on the nematodes that contribute to ΔEI values. There is no evidence from this work that the transgenic lines pose an environmental risk to the non-target nematode community. This is in contrast to the impact of the nematicides they could replace [12], [15].

The faunal analysis suggests field trials have value for assessing non-target effects on the soil nematode community. Unfortunately EU Directive 2001/18/EC [35] that governs such field trials recognises the need for field releases at the research stage (clause 23) but it does not distinguish between these and large development stage trials in the detailed data and risk assessments it demands. The EU regulations discourage small scale trials and the situation in the UK is a particular concern. The results we report are from a field trial in the UK in 2009 covered by one of only 9 consents issued in the UK from 2002-09. The total for the EU over this period is over 730 [36] in contrast to 16,049 records for USA [37] from the first field test application to the accrued total by February 2010. Both USA and Canadian regulations recognise the need for small scale experimental trials to gain the type of ecological information we have now provided. Canadian regulations identify the need to study the environmental safety and performance of the modified plants in the natural environment rather than a glasshouse [38] which our results support. Without reform, Directive 2001/18/EC will continue to hinder development of transgenic approaches that could help conserve the soil quality of productive arable fields in the EC.

## Materials and Methods

### Plasmid construction

The MDK-peptide construct comprised the promoter region of the *Arabidopsis MDK4–20* gene [20] (At5g54370) driving expression of the disulphide constrained 7-mer peptide nAChRbp (CTTMHPRLC). The peptide coding sequence was fused to an N-terminal signal sequence from the *Nicotiana plumbaginifolia* calreticulin gene to ensure efficient secretion from the roots cells as in previous work [16]. A DNA fragment encoding the signal sequence and constrained peptide sequence was obtained using a series of splice overlap extension polymerase chain reactions. Restriction enzyme sites were incorporated into the primers to allow cloning as a *Bam* HI-*Sac* I fragment into the binary vector pBI121 (Clontech, California, USA) from which the GUS coding region had been removed. The CaMV35S promoter was then excised as a *Hin*d III-*Bam* HI fragment and replaced with a 924 bp promoter region of the *MDK4–20* gene [20].

### Transformation and analysis of transgenic potato lines

The binary construct was introduced into *Agrobacterium tumefaciens* LBA4404 and leaf discs of *Solanum tuberosum* cv Désirée were transformed as described before [39]. Transgenic plantlets were rooted in liquid MS medium supplemented with 0.1 mg l^−1^ NAA before transfer to either soil or a peptide assay system as described below. Untransformed control plants were taken through identical tissue culture procedures with the exception that the *Agrobacterium* did not harbour a construct and kanamycin selection was not included.

Root exudates were collected from plant lines confirmed as transgenic by PCR analysis. Plantlets were rooted in liquid medium then transferred to 15 ml polypropylene tubes containing 7 ml sterile tap water. Plantlets were kept in a growth chamber for 14 days during which time the volume of water in each tube was maintained at 7 ml. The water was removed and used in an assay to measure the inhibition of alkaline phosphatase. For each reaction, 20 µl root exudate and 0.2 units alkaline phosphatase were preincubated for 30 min at room temperature prior to the addition of 100 µl p-nitrophenyl phosphate (1 mg ml^−1^ in 0.2 M Tris buffer). The absorbance at 405 nm was measured immediately and at subsequent 5 min intervals for one hour and the resultant regression lines used to determine enzyme activity. Four replicates were tested for each root exudate sample. Appropriate controls were included that lacked enzyme, substrate or exudate and a standard curve was derived from non-transgenic root exudate spiked with known concentrations of synthetic peptide (GL Biochemistry, Shanghai, China).

### Containment trials

The potato plants for resistance trial were grown as before [40] in a sand/loam mix containing cysts of *G. pallida* to provide an initial population of 10 viable eggs/g soil. There were 10 replicate pots for untransformed Désirée and each of three transgenic lines harbouring the MDK-peptide construct. *G. pallida* final population estimates were made for two dry soil samples of 100 g taken post harvest from each pot. Cysts were recovered from each 100 g sample using a Fenwick can, disrupted in 10 ml water and the released eggs in three replicate 1 ml aliquots counted using standard methods [41].

For nematode faunal analysis in containment, all plants were trialled in 25 cm pots containing approximately 9 kg soil taken from the future field trial site. A single potato plant or six oil seed rape (OSR; *Brassica napus*) plants were grown in each pot. Two GMNR lines studied previously that express the engineered rice cystatin OcIΔD86 either constitutively [9] or under control of a root specific promoter [10] were included in this trial. Immediately prior to planting the soil was thoroughly mixed and triplicate 150 g samples removed for nematode faunal analysis. Rooted potato plantlets taken from liquid media were transferred to a compost/Perlite mix in 7 cm pots and established for 42 days prior to transfer into the field soil. OSR was similarly pre-grown for 42 days. There were three replicate pots each for OSR, untransformed Désirée and Santé potato cultivars, two cystatin-expressing transgenic lines and three MDK-peptide transgenic lines. Plants were grown at 20±2°C on 16 h: 8 h light cycle in a containment glasshouse and a 100 g soil sample was withdrawn from the plant rhizosphere of each pot both when the untransformed cv Désirée was in flower and just before harvest. Each sample was collected from 6 positions that were 10 cm deep around a 7 cm radius from the plant stem. Soil nematodes were extracted for analysis using the Seinhorst flask method [41].

### Field trial

The field trial was conducted at the University of Leeds Field Station, Headley Hall Farm, Tadcaster, West Yorkshire, UK (DEFRA Consent 07/R31/01). This was a randomised block design with 4 replicate small plots per line using the same range of plants as in containment trial for faunal analysis. Each potato plot contained 9 plants grown from chitted tubers 38 cm apart in the row with rows 77 cm apart. OSR was planted near the site and transplanted at 17 plants/m^2^ into the replicate plots for this crop within the trial, concurrently with potato planting. The trial was surrounded with guard rows of potato cv Santé. The sampling for faunal analysis was before planting, at flowering of cv Désirée and before harvest. On the two occasions after planting, 100 g of soil was recovered from the rhizosphere of each plant and nematodes extracted from pooled samples for the nine plants per replicate plot using the tray method [41] to facilitate handling many samples. Preliminary work established that both this and the Seinhorst flask method of nematode extraction from soil provided similar values for nematode faunal analysis. Following removal of the haulms, soil samples were collected from the base of each plant upon harvest of the tubers. The soil samples from 9 plants in a replicate plot were mixed thoroughly, dried and duplicate 100 g aliquots per plot removed for *Globodera* cyst extraction and egg counts as described above.

### Bar coding of nematodes and development of genus specific qPCR primers

Soil nematodes were initially sampled from the future field trial site and extracted using the techniques described above to allow identification of the genera present. Three soil cores (2 cm diameter and 15 cm deep) were removed from each of 12 positions and the samples combined and mixed prior to nematode extraction. Each individual nematode was assigned a tentative identification based upon morphological characteristics observed using a Leica DMRB microscope. It was then digested to release DNA [25] and the lysate was stored at −20°C until required. PCR was carried out on 2 µl lysate using Biotaq Red DNA polymerase (Bioline, London, UK) and primers SSU18A (AAAGATTAAGCCATGCATG) and SSU26R (CATTCTTGGCAAATGCTTTCG) designed to conserved sequences of the SSU gene [24]. The PCR reaction conditions were as described previously [25].

PCR products were purified (QIAquick kit; Qiagen, Sussex, UK) then directly sequenced using a Taq Dye Deoxy Terminator Cycle Sequencing system (Applied Biosystems) and an automated sequencer (Applied Biosystems 373A) with primer SSU9R (AGCTGGAATTACCGCGGCTG) [24]. The resulting sequences were used in BLASTN similarity searches of GenBank nucleotide database entries allowing a putative genus to be assigned to each sample (Table 1). Multiple sequence alignments performed in MegAlign within the DNASTAR software suite (Lasergene, Madison, Wisconsin, USA) were used as a basis for designing genus specific qPCR primers (PrimerSelect, DNASTAR) to the variable regions within the SSU gene that could discriminate between the different nematode genera present at the trial site (Table 2). BLASTN searches of the GenBank database confirmed the predicted specificity of each primer pair. Genus-specific primer pairs were tested in two SYBR Green qPCR reactions with dissociation curves using DNA template prepared from a) single nematodes and b) a pool of 200 randomly selected worms. In addition, the sequence of products amplified from pools of 200 randomly selected individuals was determined. The primers were validated as specific for the range of nematodes present in the soil samples.

### qPCR based method for faunal analysis

The nematodes extracted from 100 g soil samples as described above were incubated in 1 ml of 0.25 M NaOH overnight at 25°C followed by heating at 99°C for 3 minutes. After cooling to room temperature, 200 µl 1 M HCl, 500 µl 0.5 M Tris-HCl (pH 8.0) and 250 µl 2% Triton X-100 were added and the samples heated again at 99°C for 3 min. Lysate was stored at −20°C. Comparative qPCR was performed on a Mx3005P qPCR system (Agilent Technologies, Cheshire, UK) using 5 µl template DNA in a 25 µl reaction volume with 1× iQ SYBR Green Supermix (BioRad, Herts, UK) and 300 nM of the appropriate genus specific primer pair. Reactions, including no template controls, were performed in triplicate. The reaction conditions were 95°C for 3 minutes, then 40 cycles of 95°C for 30 seconds, 60°C for 30 seconds and 72°C for 30 seconds.

Ct values obtained for a series of individuals for each genus were used to normalise mean DNA content per nematode from qPCR results, with *Aphelenchoides* as an arbitrary calibrator. For initial sampling from the field trial site, the relative numbers of nematode genera were validated with tandem measurements using a microscope-based morphological approach of nematode identification. A subsequent minor adjustment to the normalization was made after relating these parallel morphological counts to qPCR measurements of nematodes. Normalised values were used to identify relative number per genus to determine EI and SI values [22], [23] of containment and field trial soil samples.

### Statistical approaches

All analyses were carried out using SPSS version 16.0 (http://wwwspsscom/?source=homepage&hpzone=nav_bar). For Fig. 2, resistance is expressed as before [9], [40]. The number of soil samples in containment trials and the field trial is given above. The arcsin values of the proportions were analysed by the general linear model univariate procedure. This latter procedure and oneway analysis of variance was also used for the data in Fig. 4.

## Supporting Information

Figure S1 **Specificity of qPCR primer pairs for** ***Eucephalobus*** **and** ***Acrobeloides/Cephalobus*** **.** Fluorescent emission from SYBR green labeled products of individual qPCR reactions using a primer pair designed for either *Eucephalobus* (GUF + EuR) or *Acrobeloides*/*Cephalobus* (GUF + AcCR) and template DNA from each genus. For each primer pair, amplification occurred only with the correct template DNA and not with DNA from the other, closely related nematode. Primer sequences are given in Table 2.
(DOC)
