# Supplementary material for: Transgenic Potatoes for Potato Cyst Nematode Control Can Replace Pesticide Use without Impact on Soil Quality
Source: PLoS One. 2012 Feb 16;7(2):e30973. doi: 10.1371/journal.pone.0030973 (PMC3281046; doi:10.1371/journal.pone.0030973)
Supplement: Figure S1 — Specificity of qPCR primer pairs for Eucephalobus and Acrobeloides/Cephalobus . Fluorescent emission from SYBR green labeled products of individual qPCR reactions using a primer pair designed for either Eucephalobus (GUF + EuR) or Acrobeloides/Cephalobus (GUF + AcCR) and template DNA from each genus. For each primer pair, amplification occurred only with the correct template DNA and not with DNA from the other, closely related nematode. Primer sequences are given in Table 2. (DOC) [file pone.0030973.s001.doc]

**Figure S1. Specificity of qPCR primer pairs for *Eucephalobus* and *Acrobeloides/Cephalobus*.** Fluorescent emission from SYBR green labeled products of individual qPCR reactions using a primer pair designed for either *Eucephalobus* (GUF + EuR) or *Acrobeloides*/*Cephalobus* (GUF + AcCR) and template DNA from each genus. For each primer pair, amplification occurred only with the correct template DNA and not with DNA from the other, closely related nematode. Primer sequences are given in Table 2.
